# Long acting injectables: new therapeutic weapons in the battle for shortening the Duration of Untreated Psychosis

**DOI:** 10.1192/j.eurpsy.2025.2071

**Published:** 2025-08-26

**Authors:** R. De Hita Santillana, M. L. Costa Ferreira da Silva, M. del Valle Martin

**Affiliations:** 1 Hospital Universitario Jose Germain; 2 Hospital Universitario Severo Ochoa, Leganés, Spain

## Abstract

**Introduction:**

Duration of untreated psychosis (DUP) is defined as the time from manifestation of the first psychotic symptom to initiation of adequate antipsychotic drug treatment. It is associated with poorer response rates to antipsychotic medications and impaired cognition, which traslates in worse positive and negative symtomps. Various hypotheses for how untreated psychosis could impact brain function have been proposed. Dopaminergic hyperactivity leading to a progressive reduction in regional brain volumes and oxidative injury due to persistent catecholaminergic activity and prolonged activation of the hypothalamic–pituitary–adrenal axis provide possible explanations for how chronic psychosis could be neurotoxic. In addition, glutamate-mediated excitotoxicity may also contribute to these effects through neuronal overstimulation that leads to an excessive influx of calcium and subsequent excitotoxicity and, ultimately, cell death via apoptosis.

**Objectives:**

To analyze the advantages of long-acting injectable therapy in the treatment of psychotic disorders in patients with prolongued duration of untreated psychosis with a case communication.

**Methods:**

We present a 61 year old female patient, native of a rural area of Peru who moved to Spain and started treatment in our outpatient department. She does not provide any medical report. The family reports that she began to present symptoms from her first pregnancy, and has had to be admitted to hospital several times for suicide attempts. She had never taken any antipsychotic drug. During the last 30 years she developed symptoms consisting of erotomaniac and paranoid delusional ideation, cenesthetic and auditory hallucinations, soliloquy, self care deficit and disorganised speech and behaviour. She presented periods of maniac mood. As a result, she ended up isolated, taken care by his father and highly dependent on instrumental activities of daily living.

**Results:**

Treatment with Risperidone was started, although the patient presented poor adherence to it. She was finally admitted to hospital for a month and we introduced once-monthly Risperidone and Valproate with significant clinical improvement.

**Conclusions:**

Long acting injectables are a good therapeutic option in patients with psychotic disorders, even with prolongued duration of untreated psychosis.

**Disclosure of Interest:**

None Declared

